# In This Issue

**Published:** 1998

## INTRODUCTION TO ALCOHOL WITHDRAWAL

Heavy drinkers who suddenly reduce their intake of alcohol or who completely abstain from drinking frequently experience alcohol withdrawal. The manifestations of withdrawal can range from mild insomnia and irritability to serious consequences, such as seizures, hallucinations, and delirium tremens (known as DT’s). Dr. Richard Saitz reviews the mechanisms and clinical features of alcohol withdrawal and summarizes current approaches to managing the care of patients undergoing withdrawal. According to the author, the effective treatment of withdrawal symptoms can be the first step on the patient’s road to recovery from alcoholism. (pp. 5–12)

## NEUROCHEMICAL MECHANISMS UNDERLYING ALCOHOL WITHDRAWAL

During the past 25 years, researchers have made rapid progress in understanding the chemical activities that take place in the nervous system during alcohol withdrawal. At the same time, advances have been made in the clinical treatment of withdrawal, making detoxification a routine procedure that no longer is life threatening. For the most part, however, these treatment advances evolved independently from the developments in neurochemical understanding. According to Dr. John Littleton, now may be the time to rethink the goals that have been set for both the research and treatment of alcohol withdrawal. The author reviews concepts and developments in basic research on the neurochemical mechanisms underlying withdrawal. Dr. Littleton also explores the reasons why advances in basic research have not yet been translated into therapeutic gains and suggests areas that should be targeted for future research. (pp. 13–24)

## KINDLING IN ALCOHOL WITHDRAWAL

In many long-term heavy drinkers, repeated episodes of alcohol withdrawal lead to symptoms that are increasingly severe. According to Dr. Howard C. Becker, this progressive exacerbation of withdrawal may result from a “kindling” process, in which repeated alcohol abuse followed by periods of abstinence induces behavioral responses, such as seizures. Kindling in alcohol withdrawal may be caused by imbalances in brain chemicals that occur as the body attempts to adapt to the nearly constant presence of alcohol in the brain. These imbalances become more pronounced after repeated withdrawal episodes. The author describes the phenomenon of kindling and discusses some of its implications for the risk of relapse and susceptibility to alcohol-related brain damage. (pp. 25–33)

## TREATMENT OF ALCOHOL WITHDRAWAL

Mild alcohol withdrawal can cause pain and suffering; severe alcohol withdrawal can be life threatening. Drs. Hugh Myrick and Raymond F. Anton explore treatment approaches that can relieve the patient’s discomfort, prevent more serious symptoms, and limit the severity of future withdrawals. An additional benefit of withdrawal treatment is the opportunity it provides to engage patients in long-term alcoholism treatment. The authors explore the management of alcohol withdrawal and co-occurring conditions, such as depression. They also compare the different types of treatment settings, evaluate data on the effectiveness of various medications, and discuss practical considerations for dealing with specific complications, such as delirium tremens and seizures. (pp. 38–43)

## FETAL ALCOHOL SYNDROME: DOES ALCOHOL WITHDRAWAL PLAY A ROLE?

When a pregnant woman drinks alcoholic beverages, she exposes her unborn child to alcohol, placing the child at risk for a wide range of alcohol-related birth defects, including fetal alcohol syndrome (FAS). Researchers are now investigating whether alcohol withdrawal during pregnancy also contributes to the physiological and behavioral problems associated with FAS. Drs. Jennifer D. Thomas and Edward P. Riley review animal studies which suggest that altered activities of an important brain chemical (i.e., glutamate) during withdrawal may interfere with the development of nerve cells and nerve cell networks in the fetal brain. These disturbances could play a role in the behavioral deficits seen in people who were prenatally exposed to alcohol. (pp. 47–53)

## ALCOHOL HANGOVER: MECHANISMS AND MEDIATORS

People who drink to intoxication commonly experience an unpleasant collection of symptoms known as a hangover. Although relatively little research attention has focused on hangovers, current studies suggest that multiple factors contribute to the dizziness, mood disturbances, nausea, fatigue, headache, and other symptoms that characterize the condition. Drs. Robert Swift and Dena Davidson describe the hangover-promoting influences of alcohol as well as the effects of nonalcohol factors. Research suggests, for example, that some people with a high risk for alcoholism experience more severe hangover symptoms and may initiate further drinking in an effort to find relief. For other drinkers, the experience of a hangover deters subsequent heavy drinking, causing some researchers and clinicians to question the value of finding an effective hangover treatment. (pp. 54–60)

## COMPLICATIONS OF ALCOHOL WITHDRAWAL

Some patients undergoing withdrawal may experience only mild discomfort. Other patients may have medical emergencies as a result of the acute complications of withdrawal, some of which may be permanently disabling. Drs. Louis A. Trevisan, Nashaat Boutros, Ismene L. Petrakis, and John H. Krystal discuss the recognition and management of withdrawal-associated conditions such as seizures, delirium tremens, intellectual impairment, and psychiatric difficulties. The authors also present evidence to suggest that alterations in physiology, mood, and behavior may persist for months after acute withdrawal has subsided, potentially motivating relapse in abstinent alcoholics. Research is revealing the ways in which alcohol’s effects on the nervous system contribute to the processes of withdrawal. Results of this research may help improve the treatment of withdrawal and prolong abstinence. (pp. 61–66)

## DISTURBANCES OF THE STRESS RESPONSE

Stress is a ubiquitous and unavoidable experience that permeates daily life. Whether it arises from an outside source, such as a job interview or traffic accident, or from within, as with an infection or panic attack, stress evokes powerful mechanisms that enable the body to cope with these extra physiological demands. Because the stress response alters vital metabolic processes, however, powerful mechanisms also exist to regulate the level of response. Drs. Bryon Adinoff, Ali Iranmanesh, Johannes Veldhuis, and Lisa Fisher demonstrate how long-term alcohol consumption and withdrawal disrupt a key stress response system known as the hypothalamic-pituitary-adrenal (HPA) axis. The resulting imbalance in stress hormone production can alter energy metabolism, mental status, bone and muscle structure, and the body’s ability to resist infection. In abstinent alcoholics, an alcohol-impaired HPA axis may fail to protect the body from stressors that might induce relapse to drinking. Future research may lead to medications that help restore the balance of the HPA system, thus prolonging abstinence. (pp. 67–72)

## PREVALENCE, TRENDS, AND INCIDENCE OF ALCOHOL WITHDRAWAL SYMPTOMS: ANALYSIS OF GENERAL POPULATION AND CLINICAL SAMPLES

The presence of withdrawal symptoms is an indicator of a serious addiction to alcohol. In NIAAA’s Epidemiologic Bulletin No. 38, Drs. Raul Caetano, Catherine L. Clark, and Thomas K. Greenfield report the prevalence of withdrawal symptoms in three general population samples, a sample of patients in alcoholism treatment, and a sample of participants in a program for driving-under-the-influence (DUI) offenders. The data show that the prevalence of withdrawal symptoms generally was low in the general population and did not differ among ethnic groups. The prevalence of withdrawal symptoms was significantly higher in people undergoing alcoholism treatment, with rates among blacks lower than those among whites and Hispanics. The prevalence rates among DUI offenders fell between those of the general population and those of the treatment population. (pp. 73–80)

